# Differences in Immediate and Delayed Suggestibility Among Children With Dyslexia and Controls

**DOI:** 10.1002/bsl.70061

**Published:** 2026-04-24

**Authors:** Gisli Gudjonsson, Valeria Giostra, Monia Vagni

**Affiliations:** ^1^ Institute of Psychiatry, Psychology and Neuroscience King's College London London UK; ^2^ Centre for Education and Research in Forensic Psychology, The Department of Humanities University of Urbino Urbino Italy; ^3^ Department of Philosophy, Social Sciences, Humanities and Education University of Perugia Perugia Italy

**Keywords:** confabulation, delayed suggestibility, dyslexia, immediate suggestibility, resistant behavioral responses

## Abstract

The primary aim of this study was to investigate the relationship between dyslexia and suggestibility in children, and the extent to which this relationship is accounted for by performance on Word Tasks. Participants comprised 95 children with dyslexia and 109 controls. All participants completed the Gudjonsson Suggestibility Scale (GSS 2), which measures immediate and delayed suggestibility 1 week apart, five different Word Tasks, and a test of non‐verbal intelligence. Overall, the findings indicate a nuanced relationship between dyslexia and different components of suggestibility as measured by the GSS 2. This relationship is substantially mediated by poor verbal recall and difficulties in identifying absurd or implausible story content. This represents a novel contribution to scientific literature. The implications for interview practice are significant: special caution is required when interviewing children with “pure” dyslexia, as the absence of co‐morbid neurodevelopmental conditions does not preclude vulnerability during investigative interviewing or witness testimony.

## 1 Introduction

There is evidence that the relationship between suggestibility and neurodevelopmental disorders is influenced by the type of neurodevelopmental disorder. The relationship is strongest for intellectual disabilities (ID), both among adults (Clare and Gudjonsson 1993; Gudjonsson et al. 2000; Gudjonsson and Young 2021) and children (Giostra and Vagni 2024; Young et al. 2003).

Similarly, people with fetal alcohol spectrum disorders (FASD) have elevated suggestibility scores on the Gudjonsson Suggestibility Scale (Brown et al. 2011; Gilbert et al. 2024). In contrast, those with attention deficit hyperactivity disorder (ADHD) are not significantly vulnerable to leading questions and interrogative pressure, but their vulnerability relates to their disproportionate number of “don't know” replies (Gudjonsson and Young 2021), which leaves them vulnerable to delayed suggestibility when memory distrust gradually develops over time (Gudjonsson 2018). North et al. (2008) found that individuals with high functioning autism spectrum disorder (ASD) did not differ significantly from people in the general population of a similar intellect in terms of memory or suggestibility on the GSS 2.

Therefore, the type of neurodevelopmental disorder appears to have different relationships with interrogative suggestibility. In this context, an area of increasing interest for both the educational and legal community is the presence of specific learning disorders (SLD) in school‐age children. In particular, Developmental dyslexia constitutes approximately 80% of learning disabilities, making it the predominant form (World Health Organization 2016; Yang et al. 2022).

We don't have much insight into how specific learning disorders affect interrogative suggestibility despite evidence linking neurodevelopmental disorders to suggestibility.

In a nutshell, scientific research and forensic practice have highlighted the increased vulnerability of juvenile witnesses to suggestibility, as well as difficulties in language processing and information retrieval among children with dyslexia. However, there remains a limited understanding of how specific learning disabilities influence suggestibility during interrogations, despite growing evidence linking neurodevelopmental disorders to heightened suggestibility.

The aim of this study is to investigate the relationship between dyslexia and vulnerability to suggestibility, with a view to providing practical implications for the forensic field.

### 1.1 Developmental Dyslexia

Developmental dyslexia is a neurodevelopmental disorder characterized by persistent difficulties with accurate and/or fluent word recognition, poor phonological skills, and impaired spelling, despite adequate intelligence, appropriate education, and sensory abilities (DSM‐5TR; Diagnostic and Statistical Manual for Mental Disorders–Fifth Revised Edition; American Psychiatric Association 2022).

Recent studies have highlighted how diagnoses of dyslexia are on the rise and that in particular represents approximately a third of difficulties related to learning disabilities (Barbiero et al. 2019). A global systematic review estimates the prevalence of developmental dyslexia in primary school children at around 7.10% (Yang et al. 2022).

Dyslexia is often comorbid with other neurodevelopmental disorders, such as Attention Deficit Hyperactivity Disorder (Willcutt and Pennington 2000); Primary Language Impairment where the phonological deficit is the central core of both (Snowling and Hulme 2012); Dyscalculia (Moll et al. 2014) and Dyspraxia (Fawcett and Nicolson 2007).

Children with dyslexia present a distinct clinical profile characterized by significant difficulties in broader and more general cognitive abilities, such as executive functions, working memory, processing speed and language abilities (López‐Zamora et al. 2025; Alt et al. 2017).

With respect to linguistic impairments, it is widely accepted that children diagnosed with dyslexia exhibit difficulties in phonological processing (Ramus et al. 2013). Dyslexic individuals typically struggle with tasks involving phonological awareness, including phoneme manipulation, as evidenced by several studies (Bradley and Bryant 1978, 1983; Joanisse et al. 2000; Catts et al. 2005). Moreover, they tend to demonstrate weaknesses in verbal short‐term memory, as shown in assessments such as digit span and non‐word repetition tasks, according to Snowling (2000) and Szenkovits and Ramus (2005). Additionally, dyslexic children often display slow performance on rapid automatized naming tasks, which assess the speed of retrieving familiar words and their phonological representations, as indicated by studies by Denckla and Rudel (1976) and Wolf and Bowers (1999).

Furthermore, literature highlights how due to their frustration with reading, children with dyslexia are also at greater risk of developing social and emotional problems, such as anxiety, Behavioral problems, lower positive well‐being and lower levels of self‐esteem (Novita 2016; Livingston et al. 2018; Zuppardo et al. 2023).

The vulnerability to leading questions and misleading information of children with learning difficulties, specifically learning disorders such as dyslexia, has to the best of our knowledge not been researched using the Gudjonsson Suggestibility Scales, although there is substantial research on relationship of different language abilities on suggestibility and witness testimony (Perez et al. 2022).

### 1.2 Interrogative Suggestibility

Interrogative suggestibility refers to the extent to which individuals’ recollections and responses can be influenced by leading questions and interrogative pressure. According to Gudjonsson and Clark (1986, 84) model, interrogative suggestibility is defined as “the extent to which, within a closed social interaction, people come to accept messages communicated during formal questioning, as the result of which their subsequent behavioral response is affected.”

Interrogative suggestibility is measured using the Gudjonsson Suggestibility Scale (GSS; Gudjonsson 1983, 1984, 1987, 1997), which assesses how susceptible individuals are to accept leading questions (yield) and to change their answers after interrogative pressure and negative feedback (shift).

According the Gudjonsson and Clark (1986) model, three factors, must be present for a suggestible response to occur: expectations of success, uncertainty and interpersonal trust. However, merely meeting these conditions may not be sufficient to compel interviewees to comply with leading questions. In such cases, individuals can exhibit resistance to the suggestion and opt to respond with “don't know” replies instead of yielding. The model delineates two primary types of responses to leading questions and negative feedback: the “suggestible behavioral response” (SBR) involves accepting the interviewer’s suggestions, while the “resistant behavioral response” (RBR) entails rejecting the suggestions (Gudjonsson 1992, 2003; Gudjonsson et al. 2021, 2022).

RBRs are seen, according to the Gudjonsson and Clark (1986) model as a failure in discrepancy detection and inability to implement functional coping strategies when presented with leading questions and interrogative pressure to alter previous answers.

When presented with leading questions, interviewees have multiple strategies to resist yielding to the suggested information. Firstly, they can opt to provide “don't know” (DK) responses, which, despite being considered appropriate and potentially beneficial (Gudjonsson and Young 2021; Waterman and Blades 2011), may indicate difficulties with “source monitoring.” (Gudjonsson et al. 2021). This term refers to the challenge in making attributions about the origin of memory, knowledge, and beliefs (Johnson 1997; Johnson and Raye 1981; Johnson et al. 1993). Secondly, interviewees may refute the suggestion by affirming its factual inaccuracy, such as stating “that was not mentioned” or “that didn’t happen,” categorized in this study as “direct explanation” (DE) responses. Lastly, individuals can reject the suggestion outright by responding with a “no” without offering a direct justification for their inability to answer, termed as “no” reply (NO answers). These primary forms of resistant responses to leading questions during Yield 1 and Yield 2 (DK, DE, and NO answers) can be measured using the Gudjonsson Suggestibility Scales (GSS; Gudjonsson 1997; Gudjonsson and Young 2021; Gudjonsson et al. 2021, 2022; Vagni et al. 2023, 2024; Giostra and Vagni 2024; Vagni and Giostra 2024).

Recently, an additional component called “delayed suggestibility”, evaluated after a 1‐week period, has been included in the GSS 2 (Gudjonsson et al. 2016, 2020; Vagni et al. 2015, 2017, 2018). This term describes the degree to which an individual integrates misleading information, particularly from leading questions, into their later recollection, as outlined by Gudjonsson (2018, 82).

Many factors can affect the suggestibility of individuals, such as age, social and psychological traits and cognitive abilities (Ridley and Gudjonsson 2013).

### 1.3 Linguistic Abilities and Suggestibility

A rich body of literature has investigated the relationship between linguistic abilities and the child’s levels of suggestibility in children without dyslexia, but the results are often conflicting. In fact, the findings showed that specific domains of language have varying connections to children’s susceptibility to suggestion (Perez et al. 2022). About general language abilities, a lot of studies show that higher language skills are associated with lower levels of suggestibility in children between 3 and 14 years old (Boland et al. 2003; Chae and Ceci 2005; Clarke‐Stewart et al. 2004; Curci et al. 2017; Kulkofsky and Klemfuss 2008, Study 2; Roebers and Schneider 2005, Study 1; Young et al. 2003), but other studies have not found a significant correlation (Benedan et al. 2018, Study 2; Evans et al. 2014; Study 2; Gordon et al. 1993; Greenhoot et al. 1999; Roebers and Schneider 2005, Study 2).

The findings on the impact of receptive language abilities on children’s reactions to misleading questions are inconclusive (Perez et al. 2022). The studies with significant results (Curci et al. 2017; Klemfuss 2015; Kulkofsky 2010; Quas et al. 2005; Uhl et al. 2016), showed that children with better comprehension skills were less prone to suggestion, and that higher scores in receptive language were linked to reduced agreement and more precise responses to misleading questions, after adjusting for age differences.

Most of the studies that examined the relationship between expressive language abilities and children’s responses to misleading questions, found that advanced expressive language abilities were associated with decreased suggestibility (Curci et al. 2017; Danielsdottir et al. 1993; Klemfuss 2015; Melinder et al. 2005, Study 1 and 2). However, the associations were not entirely consistent across all studies.

The vulnerability to leading questions and misleading information of children with learning disorders such as dyslexia, has to the best of our knowledge not been researched using the Gudjonsson Suggestibility Scales, although it is widely accepted that children diagnosed with dyslexia exhibit linguistic impairment and there is substantial research on relationship of different language abilities on suggestibility (Perez et al. 2022).

The primary aim of the current study is to investigate the relationship between interrogative suggestibility and dyslexia and the extent to which it is mediated by different language abilities.

Following the extensive literature review of Perez et al. (2022), the primary assumption in the current study is that there is a significant relationship between learning abilities and children's witness memory and suggestibility. Furthermore, following Perez et al. (2022) findings, we explore how different domains of language (i.e., Production and comprehension, through the subtests of production of synonyms and antonyms, definitions; and detection of absurdities) are differentially related to specific vulnerabilities during questioning using the GSS 2. To make the findings more relevant to real life cases, we include children with specific learning disabilities (e.g., dyslexia) and normal controls of similar non‐verbal IQ.

### 1.4 Hypotheses

We test the following hypotheses:

Hypothesis 1 On the GSS 2, the dyslexia group will obtain lower scores on immediate and delayed recall than controls, and higher scores on immediate suggestibility, delayed suggestibility, and confabulation in memory recall.

Hypothesis 2 The dyslexia group will perform more poorly on Word Tasks despite absence of non‐verbal intellectual impairment.

Hypothesis 3 After adjusting for group, sex, age and immediate recall, the Word Tasks, particularly Word Test E (i.e., ability identify and appropriately respond to an absurd statement), will predict immediate suggestibility and delayed suggestibility.

Hypothesis 4 Out of the three resistant behavioral responses (RBRs) on Yield 1 and Yield 2, direct explanation (DE) answers will have the strongest relationship with detection of absurdities in stories.

## 2 Materials and Methods

### 2.1 Participants

In the present study, two groups were included. The first sample included 95 participants with a diagnosis of dyslexia. Their ages ranged from 9 to 16 years old (*M* = 12.55 and SD = 2.12; range = 9–16 years; 53.7% male; 46.3% female). They were recruited from several Italian assessment centers for learning disabilities. Children diagnosed with dyslexia only were included. Thus, we excluded those with diagnosed comorbidity such as dysgraphia, intellectual disabilities, attention deficit hyperactivity disorder (ADHD), and autism. In the Italian diagnostic process for Specific Learning Disorders and Dyslexia, a preliminary screening usually takes place up to the age of 6–8, but the precise diagnosis is made after the age of 8. For this reason, children over the age of 8 who had a confirmed diagnosis of Dyslexia were recruited into the sample.

The second group, used as a control group, was made up of 109 participants without language delays, dyslexia, or other developmental disorders. The children in the control group were recruited from several Italian middle and high schools (*M* = 12.58; SD = 2.17; range 9–16 years; 46.8% male and 53.2% female).

In this study only group differences in non‐verbal measure of intellectual abilities were measured.

Informed consent to participate in the study was obtained for each participant and a parent. Information was collected from the parents to verify the absence of developmental and learning disorders among the control sample and across both groups' problems with the Italian language. Among the participants, we excluded those participants who obtained a score ≤ 2 deviations from the mean on the GSS 2 memory task (i.e., outliers).

### 2.2 Instruments

#### 2.2.1 Gudjonsson Suggestibility Scale 2

The Gudjonsson Suggestibility Scale 2 (GSS 2) is a tool that traditionally measures individual levels of interrogative suggestibility (i.e., Yield 1, Yield 2, Shift, and Total Suggestibility; Gudjonsson 1997). It also measures immediate and delayed recall and confabulation in recall (Gudjonsson 1997; Clare and Gudjonsson 1993; Vagni et al. 2021).

An additional procedure standardized among children by Gudjonsson et al. (2016, 2024) provides delayed recall after a week to detect the presence of post‐event information incorporated into the original memory following the misleading information received during the previous suggestive interview. This is a measure of delayed suggestibility (Gudjonsson et al. 2024).

The answers to the misleading questions on the GSS 2 can be classified into three different types of Resistant Behavioral Responses (RBRs; Gudjonsson and Clark 1986; Gudjonsson et al. 2021, 2022; Vagni et al. 2023; Giostra et al. 2025): “no” answers, “don't know” (DK) answers, and “direct explanation” (DE) answers.

#### 2.2.2 The Word Test

The Word Test is a tool that allows you to measure different linguistic skills using some tests (Bowers et al. 2005). There is a version for children aged 6–11 (The Word test 3—Elementary, Bowers et al. 2014) and a parallel version for teenagers aged 12 and up (The Word test 2—Adolescent, Bowers et al. 2005).

*The Word Test* both Elementary and Adolescent assesses a student’s ability to recognize express critical semantic attributes in vocabulary skills, provide definitions, find antonyms, and express reasoning to detect semantic absurdities.

The Italian version of the tool (Codognotto and Magro 2012) includes 4 tasks of the original Word test with the linguistic adaptation of the items and the Italian adaptation of the Stanford–Binet Intelligence Scale Form L–M, in the Terman–Merill (Bozzo and Mansueto 1993) revision for recognition of semantic absurdities in short stories (Task E). There are 17 items in each subtest.

The tasks used in this study are:

Task A—Synonyms—This consists of the participant being asked to give a one‐word synonym for each of 17 stimulus words. (e.g., “Tell me another word for happy.” The correct answer might be “glad”). Good results on this task indicate an ability to categorize by semantic equivalence.

Task B—Semantic Absurdities—The participant is asked to identify and repair an absurd statement. There are 17 seventeen semantically absurd sentences, and the participant is asked to correct them accordingly to make them sensible (e.g., “The plumber fixes the lights. Complete it yourself so it makes sense. The correct answer might be “the pipes”).

Task C—Antonyms—The participant is asked to give a one‐word opposite for each stimulus word for example: (“Let’s do some opposites. What is the opposite of hot?”. The correct answer might be “Cold”).

Task D—Definitions—State the definition of words that include critical attributes. It is a subtest that normally has a strong correlation with “Vocabulary” of the Weschler scales (Codognotto and Magro 2012).

Task E—Absurd stories—Identify and repair an absurd statement. The child must make a judgment of absurdity or correctness by listening to short stories, motivating the possible judgment of absurdity. (e.g., “Now I’ll tell you a story, tell me what is silly in the story, or there's something wrong? e.g., “A man was found locked in his room with his hands and feet tied. He is thought to have done it alone” A possible correct answer: “There’s no way he did it alone. It must have been someone”).

The Word Test provides two main forms of language communication: (a) the production and comprehension, through the subtests of production of synonyms and antonyms, definitions (Word Tasks A, C and D); and (b) detection of absurdities (Word Tasks B and E).

#### 2.2.3 Raven Progressive Matrices

This is a well validated measure of non‐verbal measure of intellectual abilities. Participants under the age of 12 completed the Colored Progressive Matrices (CPM; Raven 1984; Belacchi et al. 2008), whilst those 12 years and over completed the Standard Progressive Matrices (Raven 1954).

### 2.3 Procedure

The same procedure was followed for all participants which involved two sessions. At the first session the Gudjonsson Suggestibility Scale 2 (GSS 2; Gudjonsson 1997) was administered following the standard procedure, except for collection of delayed recall, which was measured at 1‐week follow‐up rather than after 40–50 min delay in accordance with our previous studies (Gudjonsson et al. 2016; Vagni et al. 2015). The immediate recall relating to the GSS 2 narrative was collected, followed by the completion of the Raven Matrices administered, after which the 20 questions (interrogative procedure) commenced providing Yield 1, Yield 2, Shift and Total Suggestibility. Delayed suggestibility was measured at 1‐week follow‐up via free delayed recall of the GSS 2 narrative.

The study conformed to all ethical guidelines for research with human participants and followed the Declaration of Helsinki. The informed consent was signed before the inclusion of the children in the study, and it contained information on the objective of the study, methods of conduct, anonymity, and information on the conservation of sensitive data. The study was approved by the institutional bioethics committee of the University of Perugia (n. 19/2025; protocol 110629).

### 2.4 Analytical Strategy

Means and standard deviations were provided for continuous variables. Pearson correlations were performed to investigate the association between variables. We used Cohen's *d* (Cohen 1992) to measure effect sizes regarding the differences between the two groups (*t*‐tests): 0.20, 0.50 and 0.80 were used to detect small, medium, and large effect sizes, respectively. The corresponding Cohen's *d* effect sizes regarding the correlation coefficients were 0.10, 0.30 and 0.50.

Multivariate analysis of variance (MANOVA) was performed on the key dependent measures (All Word tasks, IR, DR, Yield 1, Yield 2, Shift, delayed suggestibility, confabulation 1 and 2, and resistant behavioral responses [RBRs]) with group (dyslexia vs. controls) as the independent (fixed) variable and the age, and sex as covariates.

Several hierarchical regression models were generated to test the predictive incremental effect of Word Tasks skills on the Yield 1, Yield 2, Shift, and delayed suggestibility scores. In a separate analysis we included DE 1 and DE 2 as the dependent variables. In all models, age, group (dyslexia vs. control), sex and immediate recall were entered in Step 1, and the five Word Tasks added in Step 2 to investigate their incremental value in predicting the independent variables. The same models were repeated for confabulation during immediate recall and delayed recall.

## 3 Results

### 3.1 Group Differences

The MANOVA showed significant main effects for Group (*F* = 10.382; [19, 182], *p* < 0.001; *η* ^2^ 0.520), and Age (*F* = 2.360; [19, 182], *p* < 0.01; *η* ^2^ 0.198). No significant main effects were found for sex.

Table 1 gives the mean scores and standard deviations of the two groups on all the tests. The results are that the two groups did not differ significantly on non‐verbal IQ or Yield 1. Regarding RBRs, a significant difference was found only for DE 2. The most significant group differences were on memory recall and the five Word Tasks, followed by Shift, total suggestibility, and confabulation, and delayed suggestibility.

**TABLE 1:** Descriptive statistics for dyslexia and control groups, *t*‐tests and effect sizes (*N* = 204).

	Dyslexia *N* = 95	Control *N* = 109	*t*	*d*
	Mean (SD; min–max)	Mean (SD; min–max)		
IQ	98.65 (9.65; 85–115)	101.18 (8.41; 85–118)	−2.00	0.27
GSS2				
Immediate recall	9.94 (4.24; 3–21)	15.39 (5.40; 6–27)	−7.94***	1.12
Delayed recall	6.40 (3.00; 2–16)	11.29 (4.66; 2–26)	−8.77***	1.25
Distortion IR	1.00 (0.99; 0–5)	0.64 (0.64; 0–2)	3.07**	0.43
Fabrication IR	0.53 (0.77; 0–4)	0.39 (0.64; 0–3)	1.43	0.20
Confabulation IR	1.51 (1.29; 0–6)	1.02 (0.97; 0–5)	3.07**	0.43
Distortion DR	0.88 (0.98; 0–3)	0.59 (0.76; 0–3)	2.44*	0.33
Fabrication DR	0.72 (0.93; 0–3)	0.61 (0.81; 0–3)	0.91	0.16
Confabulation DR	1.61 (1.47; 0–6)	1.19 (1.13; 0–5)	2.30*	0.32
Yield 1	7.58 (3.70; 0–15)	6.74 (3.18; 0–12)	1.71	0.24
Yield 2	8.73 (3.45; 0–14)	7.63 (3.48; 0–13)	2.49*	0.32
Shift	6.29 (3.31; 0–14)	4.80 (2.75; 0–11)	3.53**	0.49
Total IS	13.78 (5.49; 2–27)	11.58 (4.96; 0–22)	3.01**	0.42
Delayed suggestibility	1.03 (1.06; 0–4)	0.70 (0.88; 0–4)	2.47*	0.34
RBRs				
NO1	4.48 (2.50; 0–12)	4.66 (2.29; 0–12)	−0.53	0.08
NO2	4.14 (2.23; 1–11)	4.32 (2.23; 0–12)	−0.59	0.08
DE1	2.31 (3.33; 0–12)	2.89 (3.55; 0–13)	−1.21	0.17
DE2	1.59 (2.66; 0–10)	2.42 (3.29; 0–12)	−1.19*	0.28
DK1	0.68 (1.17; 0–6)	0.71 (1.52; 0–8)	−0.12	0.02
DK2	0.57 (1.07; 0–5)	0.62 (1.55; 0–8)	−0.29	0.04
Word test				
Task A	12.59 (2.69; 4–17)	15.67 (1.93; 8–17)	−9.50***	1.32
Task B	15.08 (2.30; 6–17)	16.34 (1.34; 8–17)	−4.84***	0.67
Task C	15.13 (2.12; 7–17)	16.17 (1.02; 12–17)	−4.60***	0.63
Task D	13.86 (2.98; 4–17)	15.12 (1.84; 7–17)	−3.67***	0.51
Task E	13.12 (2.70; 7–17)	14.93 (2.17; 7–17)	−5.30***	0.74

Abbreviations: DE 1 and 2 = Direct Explanation answers, DK 1 and 2 = Don't Know answers, DR = Delayed Recall, IR = Immediate Recall, Total IS = Total Immediate Suggestibility.

^*^ *p* < 0.05.

^**^ *p* < 0.01.

^***^ *p* < 0.001.

Differences in test scores between the dyslexia and control groups are provided in Table 1, based on *t*‐tests and Cohen's *d*.

### 3.2 Correlations With the Five Word Tasks

It is evident from Table 2 that the Word Tasks correlated most strongly with GSS 2 recall scores, immediate suggestibility, and direct explanation (DE) resistant behavioral response (RBR).

**TABLE 2:** Pearson correlations of the five word tasks with the other measures (*N* = 204).

	Word test				
	Task A	Task B	Task C	Task D	Task E
Age	0.093	0.033	0.072	0.183**	0.260***
IQ	0.109	0.217**	0.225**	0.244***	0.230**
GSS2					
Immediate recall	0.373***	0.247***	0.204**	0.289***	0.417***
Delayed recall	0.383***	0.212**	0.193**	0.247**	0.326***
Distortion IR	−0.019	0.013	0.001	0.067	−0.002
Fabrication IR	−0.077	−0.124	−0.186**	−0.148*	−0.226**
Confabulation IR	−0.061	−0.065	−0.121	−0.047	−0.139*
Distortion DR	−0.042	−0.034	0.034	0.047	0.010
Fabrication DR	0.005	−0.024	−0.003	0.037	−0.058
Confabulation DR	−0.016	−0.044	−0.019	0.054	−0.039
Yield 1	−0.283***	−0.359***	−0.267***	−0.406***	−0.467***
Yield 2	−0.349***	−0.356***	−0.223**	−0.298***	−0.449***
Shift	−0.401***	−0.319***	−0.198**	−0.316***	−0.432***
Total IS	−0.413***	−0.427***	−0.286**	−0.448***	−0.554***
Delayed suggestibility	−0.131	−0.180*	−0.114	−0.092	−0.172*
RBRs					
NO1	0.092	0.175*	0.067	0.173*	0.066
NO2	0.199**	0.166*	0.085	0.088	0.134
DE1	0.239**	0.230**	0.184**	0.279***	0.391***
DE2	0.273***	0.268***	0.163*	0.243***	0.421***
DK1	−0.050	0.029	0.103	0.053	0.081
DK2	−0.033	0.052	0.078	0.089	0.004

Abbreviations: DE 1 and 2 = Direct Explanation answers, DK 1 and 2 = Don't Know answers, DR = Delayed Recall, IR = Immediate Recall, Total IS = Total Immediate Suggestibility.

^*^ *p* < 0.05.

^**^ *p* < 0.01.

^***^ *p* < 0.001.

### 3.3 Correlations of Delayed Suggestibility With the Other Measures

Table 3 shows general consistency in the correlations across the two groups. Delayed suggestibility did not correlate significantly with non‐verbal IQ, age, immediate and delayed recall, or Shift. It correlated significantly with Yield 1, Yield 2, total suggestibility, and confabulation on delayed recall, and Task B of the Word test, all with low effect sizes.

**TABLE 3:** Pearson correlations between delayed suggestibility and GSS 2, RBRs and word tasks (*N* = 204).

	All sample *N* = 204	Dyslexia *N* = 95	Control *N* = 109
Age	−0.040	−0.050	−0.029
IQ	−0.047	−0.072	0.034
GSS2			
Immediate recall	−0.165*	−0.144	−0.059
Delayed recall	−0.156*	−0.152	−0.037
Confabulation IR	0.107	0.074	0.072
Confabulation DR	0.249**	0.248*	0.200*
Yield 1	0.278***	0.273**	0.251**
Yield 2	0.238**	0.180	0.258**
Shift	0.087	0.043	0.055
Total IS	0.238**	0.232*	0.185*
RBRs			
NO1	−0.130	−0.179	−0.065
NO2	−0.079	−0.052	−0.097
DE1	−0.202**	−0.172	−0.213*
DE2	−0.198**	0.143	−0.216*
DK1	0.025	−0.026	0.072
DK2	−0.049	−0.139	0.018
Word tasks			
Task A	−0.131	−0.089	0.023
Task B	−0.180*	−0.238*	0.049
Task C	−0.114	−0.149	0.112
Task D	−0.092	−0.194	0.189*
Task E	−0.172*	−0.162	−0.070

Abbreviations: DE1 and 2 = , DK 1 and 2 = Don't Know answers, Total IS = Total Immediate Suggestibility.

^*^ *p* < 0.05.

^**^ *p* < 0.01.

^***^ *p* < 0.001.

### 3.4 Hierarchical Regression Models

Several hierarchical regression models were generated to test the predictive effect of Word Tasks skills on the Yield 1, Yield 2, Shift, and delayed suggestibility scores (Table 4). In a separate analysis we included DE 1 and DE 2 as the dependent variables (Table 5). In all models, age, group (Dyslexia vs. Control), sex and immediate recall were entered in Step 1, and the five Word Tasks in Step 2 to investigate their incremental value predicting the independent variables.

**TABLE 4:** Hierarchical linear regression models of the age, sex, immediate recall, and word test tasks on immediate and delayed suggestibility scores (*n* = 204).

Predictor	Yield 1		Yield 2		Shift		Delayed suggestibility	
	*B*	*β*	*B*	*β*	*B*	*β*	*B*	*β*
Step 1								
Intercept	12.88		15.78		11.27		1.60	
Age	−0.26	−0.16*	−0.33	−0.20**	−0.19	−0.13*	−0.01	−0.02
Sex	−0.10	−0.02	−0.58	−0.08	−0.13	−0.02	−0.05	−0.02
Group	0.56	0.08	0.26	0.04	−0.43	−0.07	−0.23	−0.12
IR	−0.25	−0.41***	−0.24	−0.38***	−0.19	−0.35***	−0.02	−0.10
	*R* ^2^ = 0.191***		*R* ^2^ = 0.218***		*R* ^2^ = 0.186***		*R* ^2^ = 0.039	
Step 2								
Intercept	19.67		21.98		14.76		2.43	
Age	−0.15	−0.10	−0.26	−0.16*	−0.13	−0.09	−0.01	−0.01
Sex	−0.02	−0.01	−0.34	−0.05	0.09	0.01	−0.04	−0.02
Group	1.31	0.19*	1.43	0.20**	0.89	0.16*	−0.17	−0.09
IR	−0.19	−0.30***	−0.18	−0.29***	−0.14	−0.25**	−0.01	−0.08
Task A	−0.03	−0.02	−0.24	−0.20**	−0.28	−0.25**	0.01	0.03
Task B	−0.29	−0.16*	−0.40	−0.22**	−0.20	−0.12	−0.08	−0.15
Task C	0.10	0.05	0.13	0.06	0.23	0.13	−0.00	−0.01
Task D	−0.17	−0.12	0.17	0.12	0.03	0.03	0.03	0.09
Task E	−0.31	−0.23**	−0.34	−0.25**	−0.28	−0.23**	−0.04	−0.10
	*R* ^2^ = 0.330***		*R* ^2^ = 0.346***		*R* ^2^ = 0.302***		*R* ^2^ = 0.061	
	Δ*R* ^2^ = 0.139***		Δ*R* ^2^ = 0.128***		Δ*R* ^2^ = 0.126***		Δ*R* ^2^ = 0.022	

*Note:* Sex: 1 = male, 2 = female; Group: 1 = Dyslexia, 2 = control.

Abbreviation: IR = Immediate Recall.

^*^ *p* < 0.05.

^**^ *p* < 0.01.

^***^ *p* < 0.001.

**TABLE 5:** Hierarchical linear regression models of the age, sex, immediate recall, and word test tasks on DE 1 and DE 2 scores (*n* = 204).

Predictor	DE 1		DE 2	
	*B*	*β*	*B*	*β*
Step 1				
Intercept	−3.12		−4.54	
Age	0.34	0.21**	0.31	0.22**
Sex	−0.16	−0.02	0.42	0.07
Group	−0.45	−07	−1.27	−0.19*
IR	0.19	0.31***	0.19	0.36***
	*R* ^2^ = 0.144***		*R* ^2^ = 0.203***	
Step 2				
Intercept	−7.35		−7.87	
Age	0.25	0.16*	0.24	0.17**
Sex	−0.29	−0.04	0.29	0.05
Group	−1.36	−0.20**	−0.98	−0.16**
IR	0.14	0.22**	0.15	0.27***
Task A	0.14	0.11	0.13	0.12
Task B	0.11	0.06	0.22	0.14
Task C	−0.05	−0.02	−0.13	−0.08
Task D	0.03	0.03	−0.14	−0.12
Task E	0.35	0.27**	0.34	0.29**
	*R* ^2^ = 0.226***		*R* ^2^ = 0.291***	
	Δ*R* ^2^ = 0.082**		Δ*R* ^2^ = 0.088***	

*Note:* Sex: 1 = male, 2 = female; Group: 1 = Dyslexia, 2 = control.

Abbreviations: DE 1 and DE 2 = Direct Explanation, IR = Immediate Recall.

^*^ *p* < 0.05.

^**^ *p* < 0.01.

^***^ *p* < 0.001.

The results in Table 4 show that adding the Word Tasks in Step 2, added 13%–14% to the variance in Yield 1 and Yield 2, and Shift. Task B added significantly to the variance in Yield 1 and 2; Task E added significantly to the variance in Yield 1, Yield 2, and Shift. In contrast, Task A proved significant for Yield 2 and Shift. The models were not significant for delayed suggestibility. Tasks C and D did not assume significance.

Table 5 shows the results of the regression models for DE 1 and DE 2. In Step 1, age, Group, and immediate recall accounted for 14.4% and 20.3% in the variance in DE 1 and DE 2, respectively. In Step 2, the Word Tasks added 8% and 9% to the variance in DE 1 and DE 2 and Group variable increased its effects.

We repeated the same regressions for confabulation on both immediate and delayed recall, separately, as the independent variable. Only Group was significant a predictor of confabulation on IR (*β* = −17; *p* < 0.05; *R* ^2^ = 0.082) and confabulation on DR (*β* = −21; *p* < 0.05; *R* ^2^ = 0.074).

## 4 Discussion

The primary aim of the current study is to investigate the relationship between interrogative suggestibility and dyslexia and the extent to which it is mediated by different language abilities.

Previous studies have found a significant relationship between witnesses’ learning abilities and memory and children’s suggestibility (Perez et al. 2022). The present study aimed to fill the gap in understanding how specific learning disabilities influence suggestibility during interrogations.

We explored how different linguistic domains (i.e., production and comprehension, through the subtests of synonym and antonym production, definitions, and nonsense sentence detection) are differentially related to specific vulnerabilities during interrogations using the GSS 2. To make the results more relevant to real‐world cases, we included children with specific learning disabilities (e.g., dyslexia) and normal controls with similar nonverbal IQs.

Comparisons between the two groups (Dyslexia vs. Control Groups) highlighted how children with Dyslexia showed lower performance on memory tasks and higher levels of suggestibility. They also showed a greater tendency to produce memory errors, such as distortion, fabrication, and confabulation, and poorer language skills (see Table 1).

Greater language skills, as measured by the Word Test Tasks, showed positive correlations with more accurate and detailed Immediate and Delayed Recall and with Resistant Behavioral Responses with high discrepancy detection, and significant negative correlations with immediate and delayed suggestibility scores (see Tables 2 and 3).

The hierarchical regression models demonstrated that immediate recall on the GSS 2 narrative, followed by Word Tasks, were the two largest contributory factors to the variance in immediate suggestibility. The contribution of group (dyslexia vs. controls) to the total variance in Step 2, as an independent predictor variable of Yield 1, Yield 2, and Shift was significant but modest. What appears to be more important than the dyslexia diagnosis per se in predicting immediate suggestibility, is poor recall of the GSS narrative combined with Word Tasks deficits, particularly on Task E. This is a novel finding.

The robust finding regarding Word Task E across Yield 1, Yield 2 and Shift indicates that poor judgment of story absurdity is an impactful factor on immediate suggestibility. This finding, combined with Word Task E being the only significant predictor of direct explanation answers on Yield 1, supports the relationship between immediate suggestibility and failure in *discrepancy detection* (Gudjonsson 2003; Schooler and Loftus 1986). Based on the article of Schooler and Loftus (1986) the two main factors affecting discrepancy detection in an interrogative suggestibility context are strength of the original information in memory and the way the post‐event information is presented.

Gudjonsson (2003) points out that failure in discrepancy detection may be necessary for yielding to leading questions, but it is not sufficient to result in a Yield reply. Interviewees may simply give DK answers. DK answers are the weakest resistant behavioral responses to suggestions (Gudjonsson et al. 2024). It is also important that some Yield and Shift answers may represent conscious compliance rather than internalization of misleading information (Polczyk et al. 2024).

In contrast to DK replies, DE answers are good examples of discrepancy detection where an explanation is provided as to why the misleading question is not accepted. This overlaps theoretically with failure to detect absurdities in stories as measured by Word Task E, which does not apply to No and DK answers. It supports Gudjonsson's (2003, 2018) argument that DK answers during immediate suggestibility are a failure in discrepancy detection. It suggests that whilst not accepting the expectation component of the misleading question DK answers can over time and in certain circumstances lead to memory distortions, confabulation, and false statements. It represents a potential vulnerability factor to delayed suggestibility, particularly when it leads to profound memory distrust (Gudjonsson 2018). It is commonly found in people with attention deficit hyperactivity disorder (ADHD; Gudjonsson et al. 2007; Gudjonsson and Young 2021).

The current findings corroborate previous research of a modest correlation between immediate and delayed suggestibility (Gudjonsson et al. 2016, 2024; Wachi et al. 2019). A regression model found that there were no significant overall effects of the immediate suggestibility and Word Task measures on delayed suggestibility. The unique feature of delayed suggestibility, as measured by the GSS 1 and 2, is that the content of the misleading questions is incorporated into memory recollection at 1 week follow‐up. This requires the misleading content to be subsequently recalled, implying memory capacity and active reconstruction of original story. This suggests a complicated relationship between memory and delayed suggestibility, which may explain why no significant correlation was found in the current study between delayed recall and overall correct recollection of the GSS 2 narrative content.

Delayed suggestibility, as measured by the GSS, in contrast refers to errors introduced into the narrative by the misleading questions (i.e., they are provoked errors). Unlike immediate suggestibility, delayed suggestibility was not significantly related to immediate or delayed recall on the GSS 2 narrative. This is likely to be related to the fact that certain memory capacity is required to consolidate and retrieve the misinformation from the misleading questions. The child needs to retain the misleading information over time to incorporate it into the narrative at 1 week follow‐up. What we know from one of our previous studies (Gudjonsson et al. 2020), is that delayed suggestibility is strongly associated with severity of trauma symptoms. In fact, trauma symptoms accounted for 43.4% of the variance in delayed suggestibility after controlling for age, sex, IQ, and immediate recall. This may help explain why delayed suggestibility plays such a powerful part in internalized false confessions among suspects detained in custody and pressured during questioning (Gudjonsson 2003, 2018). In case of child victims of sexual abuse, it is the trauma associated with the abuse itself that they are questioned about that exacerbates their susceptibility to delayed suggestibility (Giostra et al. 2025).

Overall, our findings support Perez et al. (2022) comprehensive review that there is a “nuanced relationship” between language abilities and children's vulnerabilities to suggestions, whether in the form of leading questions or interrogative pressure. The current findings add to the available scientific literature by focusing on children with dyslexia. Despite absence of other co‐morbid developmental problems (e.g., ADHD and ASD), their most prominent vulnerabilities to witness testimony, as measured by GSS 2, are their poor recall for the narrative, problems with identifying bizarre story content, and proneness to confabulation in recall. The implication for interview practice is that special caution is required when interviewing children with “pure” dyslexia, because absence of co‐morbid neurodevelopmental problems still leaves them vulnerable during witness testimony.

There were some limitations. First, the inclusion in the current study of the dyslexia participants was based on available data from the assessment centre where they attended. No independent diagnostic procedure was applied for the purpose of the current study. In future research it would be important to directly compare groups of children with different types of neurodevelopmental disorders and the incremental impact of additional co‐morbid problems on the reliability of witness testimony. Despite this limitation the current findings are robust and important. Second, only group differences in non‐verbal measure of intellectual abilities were measured. The extent to which the two groups may have differed in verbal intellectual abilities is unknown.

In conclusion, the main finding is that there is a nuanced relationship between dyslexia and different scores on the GSS 2, which is substantially mediated by poor verbal recall and difficulties in identifying absurd story content.

## Author Contributions

**Gisli Gudjonsson:** validation, formal analysis, investigation, data curation, writing – original draft preparation, writing – review and editing. **Valeria Giostra:** conceptualization, methodology, writing – original draft preparation, writing – review and editing. **Monia Vagni:** conceptualization, methodology, validation, formal analysis, investigation, data curation, writing – original draft preparation, writing – review and editing All authors have read and agreed to the published version of the manuscript.

## Funding

The authors have nothing to report.

## Ethics Statement

The study was conducted in accordance with the Declaration of Helsinki, and approved by the institutional bioethics committee of the University of Perugia (Minute 19/2025; protocol 110629, 12 May 2025).

## Consent

Informed consent was obtained from all subjects involved in the study.

## Conflicts of Interest

The authors declare no conflicts of interest.

## Data Availability

The data presented in this study are available on request from the corresponding author.
